# Implementation of early rehabilitation for critically ill children in China: A survey and narrative review of the literature

**DOI:** 10.3389/fped.2022.941669

**Published:** 2022-08-12

**Authors:** Ting Zhang, Xiaoling Duan, Ying Feng, Wei Jiang, Xueqin Hou, Ling Liu, Qinrong Huang, Xiang Tang, Li Lin, Mingqiang Zhang, Liang Tao, Guoqing Liu, Yuxia Chen, Nong Xiao

**Affiliations:** ^1^Department of Rehabilitation, Children's Hospital of Chongqing Medical University, Chongqing, China; ^2^National Clinical Research Center for Child Health and Disorders, Chongqing, China; ^3^Ministry of Education Key Laboratory of Child Development and Disorders, Chongqing, China; ^4^Chongqing Key Laboratory of Pediatrics, Chongqing, China

**Keywords:** early rehabilitation, critical illness, child, tertiary care centers, survey

## Abstract

**Introduction:**

The focus of this survey was to understand the current status of implementation of early rehabilitation for critically ill children in China. We also reviewed the available literature on this topic for further insights to inform its future development.

**Materials and methods:**

We used a cross-sectional study design to survey tertiary hospitals nationwide. Questionnaires were distributed *via* the social media platform “WeChat Questionnaire Star” within the framework of the Rehabilitation Group of the Pediatrics Branch of the Chinese Medical Association. A narrative literature review on the implementation of the early rehabilitation for critically ill pediatric and/or adult patients was carried out.

**Results:**

A total of 202 valid questionnaires were received. About half (*n* = 105, 52.0%) of respondent hospitals reported that they implement early rehabilitation for critically ill children. Among these 105 hospitals, 28 implemented a continuous chain of early rehabilitation. A total of 24 hospitals had set up permanent specialized centralized early rehabilitation units for critically ill children.

**Implications and future directions:**

Early rehabilitation for critically ill children is not widely available in China and only a minority of hospitals implement a continuous chain of early rehabilitation. To improve this undesirable situation, we suggest creating a two-level integrated system comprising centralized early rehabilitation units and surrounding early rehabilitation networks within a region.

## Introduction

With the development of critical care medicine, an increasing number of children have survived critical illness in the past few decades (1, 2). As a result of critical illness, these children are at risk of physical, neurocognitive and psychosocial sequelae (3, 4), which can lead to lifelong impediments and reduced quality of life, not only for the affected child but also for the whole family (4–6). The increasing number of young survivors with moderate or severe disabilities poses a great challenge to rehabilitation professionals. Several models of rehabilitation for critical illness have been proposed. To date, these models have concentrated on early rehabilitation, which is commenced in the intensive care unit/pediatric intensive care unit (ICU/PICU), continued in the acute care ward and terminated at discharge from the acute care hospital (7, 8). However, relevant literature focusing on these children is sparse (9).

Despite China's massive and urgent clinical needs, little is written about the scope of early rehabilitation for critically ill children. Some initial practice has taken place in a few hospitals in the last 5 years, but its development is unfocused and uneven with limited theoretical support and consideration. The focus of our survey was to understand the current status of implementation of early rehabilitation for critically ill children in China. We also reviewed the available literature on early rehabilitation for critically ill patients, to further illuminate the topic and inform future development.

## Materials and methods

### Study design and participants

The survey used a cross-sectional observational study design to survey Tertiary care centers nationwide in China. Questionnaires were distributed to directors of the related departments within the framework of the Rehabilitation Group of the Pediatrics Branch of the Chinese Medical Association. Because no director of the related departments of hospitals in the provinces/cities of Hong Kong, Macau and Taiwan were in the Rehabilitation Group, so no questionnaire was expected from these provinces.

Only tertiary care centers were surveyed, considering that the implementation of early rehabilitation for critically ill children is still in the initial stage in China. Tertiary care centers in China are those of the highest level and defined as cross-regional, providing comprehensive and specialized medical care with extensive medical education and research functions (10).

### Survey instruments and data collection procedures

A questionnaire was developed based on the authors' direct field experiences. To ensure content validity, the draft questionnaire was completed and evaluated by experts in several hospitals and was revised based on their suggestions. The final questionnaire was sent to directors of the related departments *via* the social media app “WeChat Questionnaire Star.”

The questionnaire consisted of five sections: respondent, department and hospital information and four key components of early rehabilitation for critically ill children: (1) the status of implementation and planning, (2) the form and scale of implementation, (3) treatment capacity and (4) medical staff. The items included yes/no and multiple-choice responses, as well as free text options. An additional file shows the questionnaire (English translation version) in detail (Appendix 1).

Each returned questionnaire was carefully reviewed for completeness and consistency. For questionnaires with any incomplete responses or suspected errors, data were confirmed by WeChat contact or telephone call. If no answer could be obtained, that questionnaire was excluded as a “no response.”

### Statistics and data analysis

The data were entered into a database using Microsoft Excel (Microsoft Corp, Redmond, WA) software and cleaned, checked and analyzed using SPSS statistical software for Windows, version 21.0 (Chicago, IL, USA). Descriptive statistics were used to present the data. Nominal data are reported as proportions. Comparisons of the proportions were made between different types of hospitals using the chi-square test or Fisher's exact test. The level of statistical significance was established at *p* <0.05.

### Search strategy

A narrative literature review was carried out by searching PubMed with the search words “intensive care unit,” “critical illness,” “critically ill,” “children,” “child,” “pediatric,” “early rehabilitation,” “early mobilization,” “rehabilitation,” “continuous chain,” “continuum,” “centralized rehabilitation,” “specialized rehabilitation,” “sub,” “critical,” and “acute.” The data search was further augmented by scanning the references of identified papers. We review and analyzed these studies for further insights to inform the future development of the early rehabilitation for critically ill children in China.

## Results

**Figure 1:**
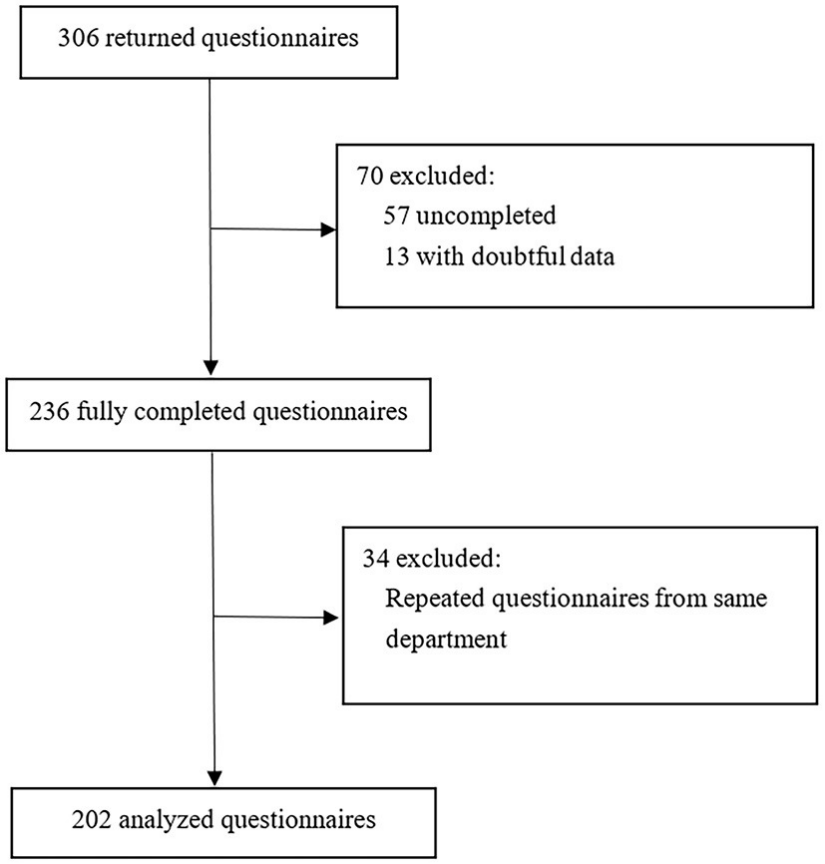
Study flow chart.

Between March 25, 2021, and April 7, 2021, 306 returned questionnaires were received, of which 104 were excluded (57 were excluded due to uncompleted information, 13 with doubtful data, and 34 due to repeated questionnaires from the same department). The data were judged to be doubtful mainly because of the logical contradiction among the answer to several questions in one questionnaire or the obvious mistakes based on common sense, e.g., the number of beds in the specialized centralized early rehabilitation units was over 100. For the repeated questionnaires from the same department, only the questionnaires from the chief director of a department were kept. If there is no first choice, questionnaires from the director of the specialized centralized early rehabilitation unit within a certain department as the second-best choice (Figure 1). This left 202 valid questionnaires from all expected provinces and cities in China. Of the 202 respondent hospitals, 134 (66.3%) were general hospitals, 26 (12.9%) were children's hospitals and 42 (20.8%) were maternal and child health hospitals.

### The status of implementation and planning

The status of implementation and planning of early rehabilitation for critically ill children at the time of the survey is shown in Table 1. About half (*n* = 105; 52.0%) of respondent hospitals reported that they implement early rehabilitation for critically ill children; the percentage for children's hospitals was as high as 88.5%, but for general hospitals and maternal and child health hospitals were 44.8 and 52.4%, respectively. The pairwise comparisons were significant between the children's hospitals and general hospitals (χ^2^ = 16.646, *p* <0.001) and between children's hospitals and maternal and child health hospitals (χ^2^ = 9.340, *p* = 0.002), but not significant between general hospitals and maternal and child health hospitals (χ^2^ = 0.743, *p* = 0.389). Similarly, a pairwise comparison of the number of hospitals that had not implemented early rehabilitation for critically ill children but planned to implement it in the next 3 years was significant between children's hospitals and general hospitals (χ^2^ = 10.956, *p* = 0.001) and between children's hospitals and maternal and child health hospitals (χ^2^ = 6.725, *p* = 0.010), but not significant between general hospitals and maternal and child health hospitals (χ^2^ = 0.491, *p* = 0.484). Only one (3.8%) children's hospital had no plan to implement rehabilitation of critically ill children in the next 3 years, whereas 18 (13.4%) general hospitals and 5 (11.9%) maternal and child health hospitals had no such plan.

**Table 1:** The status of planning and implementation of rehabilitation for critically ill children.

	**General hospitals (*n* = 134)**	**Children's hospitals (*n* = 26)**	**Maternal and child health hospitals (*n* = 42)**	**Total (*N* = 202)**
Already implemented	60 (44.8)	23 (88.5)	22 (52.4)	105 (52.0)
Not yet implemented but plan to implement	56 (41.8)	2 (7.7)	15 (35.7)	73 (36.1)
Not yet implemented and no plan	18 (13.4)	1 (3.8)	5 (11.9)	24 (11.9)

*Values are presented in number and percentage in brackets*.

### The form and scale of implementation

Among the 105 hospitals that implemented early rehabilitation for critically ill children, 28 (26.7%) hospitals implemented a continuous chain of early rehabilitation that commenced in the ICU/PICU and continued in the acute care ward when patients were discharged from the ICU/PICU, 65 (61.9%) hospitals implemented early rehabilitation only in the ICU/PICU, and 12 (11.4%) hospitals implemented early rehabilitation only in the acute care ward. In addition, among the 28 hospitals implementing a continuous chain of early rehabilitation, 23 hospitals had permanent centralized early rehabilitation units specifically designed for critically ill children, and the other 5 hospitals only provided temporary bed units for critically ill patients when needed. Meanwhile, among the 12 hospitals implementing early rehabilitation only in the acute care ward, only 1 hospital had a permanent centralized early rehabilitation unit, and the other 11 hospitals would provide temporary bed units for these children when needed. So, a total of 24 hospitals had built permanent centralized early rehabilitation units for critically ill children.

Of the 24 early rehabilitation units, 22 (91.7%) had fewer than 20 beds and 18 (75.0%) had 10 or fewer beds; 22 (91.7%) accepted fewer than 100 patients per year and 17 (70.8%) accepted fewer than 50 patients per year.

### Treatment capacity

Table 2 shows the treatment capacity of the 24 early rehabilitation units. All had the capacity to deal with neurological injury caused by infections, trauma and other pathogenesis. Most of them could accept patients after central nervous system tumor or epilepsy surgery, but only some could accept patients after orthopedic or cardiac surgery.

**Table 2:** Treatment capacity of 24 specialized centralized units implementing early rehabilitation for critically ill children.

	**No. “yes”**	**%**
**Disease entity**
Acquired neurological injury	24	100.0
Post nervous system tumor surgery	20	83.3
Post epilepsy surgery	15	62.5
Post orthopedic surgery	10	41.7
Post cardiac surgery	9	37.5
**Type of dysfunction**
Motor dysfunction	24	100.0
Disturbance of consciousness	22	91.7
Swallowing dysfunction	23	95.8
Respiratory dysfunction	16	66.7
Bladder or urethral dysfunction	18	75.0
**Supportive equipment** ***in situ***
Tracheal tube	15	62.5
Nasogastric tube	23	95.8
Jejunum tube	13	54.2
Indwelling catheter	21	87.5
Peripherally inserted central catheter	17	70.8
Central venous catheter	9	37.5
Ventilator	7	29.2
**Indications for transfer out of rehabilitation unit**
Seizures occurring several times a day	4	16.7
Seizures occurring > 10 times a day	7	29.2
Status epilepticus	18	75.0
Pneumonia	3	12.5
Severe pneumonia	16	66.7
Respiratory failure	20	83.3
Cardiac failure	21	87.5
Intracranial hypertension	14	58.3

The treatment of motor dysfunction, disturbance of consciousness and swallowing dysfunction were basic skills mastered by nearly all the 24 units. Respiratory and bladder/urethral dysfunction were managed by the majority.

Patients with a nasogastric tube or an indwelling catheter were accepted by nearly all the 24 units; 15 units accepted patients with a tracheal tube and 7 units accepted ventilated patients.

In the event of deterioration of condition or the development of indications requiring additional medical care, most of the 24 units indicated they were able to deal with the occasional occurrence of seizure in a patient, but would transfer patients to a suitable ward or unit if they developed status epilepticus; similarly, patients with pneumonia were usually treated within the unit but were transferred to a suitable ward or unit if they developed severe pneumonia. Respiratory failure and cardiac failure were also indications for transferring patients for most of the units.

### Medical staff

Among the 24 units that responded, 15 had independent workgroups comprising doctors, nurses and therapists, and one unit had an independent group of doctors. All the units indicated that they invited doctors from other departments for consultations, including ICU/PICU, neurology, neurosurgery, respiratory medicine, orthopedics and nutrition, et al.

The three most frequently involved doctor specialists were those from pediatrics/pediatric surgery, rehabilitation medicine and neurology/pediatric neurology. The three most frequently involved types of the therapist were from the fields of rehabilitation therapeutics, sports medicine and traditional Chinese medicine.

## Discussion

This study provides an initial descriptive analysis of the practice characteristics of early rehabilitation of critically ill children in China. The study has several strengths and to our knowledge, this is the first and largest practice analysis on this topic published to date. Responders spanned almost all provinces and cities in China. As mentioned above in Section Study design and participants, no questionnaire was expected from the provinces/cities of Hong Kong, Macau and Taiwan because of the objective limitation of the questionnaire distribution. Valid questionnaires were obtained from all other provinces/cities in China. The survey responses illuminated some notable facts: early rehabilitation for critically ill children is not widely available in China—only about half of the respondent hospitals reported that they implement it, and only a minority of hospitals implement a continuous chain of early rehabilitation that commences in the ICU/PICU and continues in the acute care ward. Specialized centralized early rehabilitation units for critically ill children were even sparser. The survey was carried out within the framework of the Rehabilitation Group of the Pediatrics Branch of the Chinese Medical Association and only tertiary care centers were surveyed; this gave a concentrated view of the situation, and in reality, far fewer than half of the hospitals are capable of implementing the early rehabilitation of critically ill children and most of the existing early rehabilitation is incomplete. Compared to the rate (77.8%) of the implementation of early mobilization within PICUs in Canada according to a national survey conducted 9 years ago (11), the gap in this field is huge between China and Canada. To help improve this undesirable situation, we reviewed the available literature and analyzed practical solutions as follows.

Currently, “early rehabilitation in ICU” in the literature and in practice frequently refers to exclusive mobility or exercise programs (12), despite early rehabilitation in the ICU is a strategy for whole-body rehabilitation and accomplished by interruption of sedation, protocol-driven spontaneous breathing trials, and physical and occupational therapy (13). The latest evidence suggests that early mobilization in ICU is safe, efficacious and cost-effective in critically ill adults, and at least as safe and feasible in critically ill children (3, 13–17). As a result, early mobilization, as a key aspect of the ABCDEF bundle, has been widely accepted academically and should be implemented into everyday practice in both the ICU and PICU (15, 18, 19).

Nevertheless, our survey results suggested that the implementation of early rehabilitation in ICU/PICU is not ideal. Only about half of respondent hospitals reported that they can implement early rehabilitation for critically ill children, and a few of them could only implement early rehabilitation after patients were discharged from the ICU/PICU rather than while they were admitted there. We believe that the usual working framework and protocols of hospitals in China are the reason for this, as rehabilitation staff outside of the ICU/PICU must be formally consulted by ICU/PICU physicians to work with critically ill patients. However, ICU/PICU physicians are often focused on saving the life of the critical patient and may not be aware of patients' loss of functioning and need for rehabilitation. This problem also exists in PICUs in developed countries. Several studies in Europe and North America have revealed that the major barrier to early mobilization in PICUs is the lack of physician order (11, 19, 20).

Therefore, to promote early mobilization in the ICU/PICU in China, we must promote the concept of early mobilization to ICU/PICU physicians. We suggest that rehabilitation departments and ICU/PICUs foster ongoing interdisciplinary cooperation, including holding multidisciplinary ward rounds and communicating academic knowledge regularly.

Several studies have further shown that besides early rehabilitation commenced in the ICU, a continuous chain of early rehabilitation on the acute care ward can also lead to an earlier discharge from the hospital and improved functional recovery and is also cost-effective and safe for adult survivors of critical illness. The results of these studies are summarized in Table 3 (7, 8, 21–25).

**Table 3:** Literature on a continuous chain of early rehabilitation on the acute care ward.

**References, country**	**Study type**	**Patient population**	**Group allocation**	**Number**	**Description of therapy**	**Safety**	**Efficacy**	**Cost-effectiveness**	**Feasibility**	**Shortened hospital stays**	**Patient/family satisfaction**
Berney et al. (7), Australia	Cohort study	Survivors of critical illness without major disorders of the central nervous system and long-bone or unstable fractures.	Intervention Control	74 76	Received protocolized rehabilitation program that commenced in the intensive care unit (ICU) and continued on the acute care ward, and which continued for a further 8 weeks following hospital discharge as an outpatient program. Received standard care.	√	–	–	–	√	–
Gruther et al. (8), Austria	Prospective randomized controlled study	Survivors of critical illness after discharge from the intensive care unit	Intervention Control	24 29	Received an early rehabilitation program on general ward. Received physical therapy as ordered by the primary care team on general ward.	√	√	√	–	√	–
Andelic et al. (21), Norway	Prospective cohort study	Severe traumatic brain injury (TBI) patients	Intervention Control	31 30	Received continuous chain of rehabilitation that began with the acute phase. Received broken chain of rehabilitation that started in the subacute phase of TBI.	–	√	–	–	–	–
Engberg et al. (22), Denmark	Cohort study	Severe TBI	Intervention Control	39 21	Received centralized subacute rehabilitation in a regional brain injury unit. Received subacute rehabilitation in different wards.	–	√	–	–	–	–
Andelic et al. (23), Norway	Prospective cohort study	Severe TBI	Intervention Control	30 29	Received continuous chain of rehabilitation that began with the acute phase. Received broken chain of rehabilitation that started in the subacute phase of TBI.	–	–	√	–	–	–
Wang et al. (24), China	Randomized feasibility study	Severe stroke-associated pneumonia	Intervention Control	12 12	Received additional early and follow-up rehabilitation exercises and correct rehabilitation education. Received usual care without integrated management.	–	√	–	√	√	√
Wijnen et al. (25), Netherlands	Prospective, non-randomized controlled clinical study	Multi-trauma	Intervention Control	65 67	Received integrated multi-trauma rehabilitation service approach including earlier transfer of patients to a specialized trauma rehabilitation unit. Received usual care.	–	–	√	Inconclusive	–	–

However, in our survey, among the 105 hospitals claiming to be able to implement early rehabilitation for critically ill children, only 40 could immediately implement early rehabilitation in acute care wards after patients' discharge from the ICU/PICU. Other hospitals may be limited by a lack of technology and professionals. There is often a large time gap between acute care and inpatient rehabilitation—the so-called “rehab hole” mentioned by Simmel et al. (26, 27). They further proposed that specialized rehabilitation facilities are established to fill this gap (26, 27). In our survey, only 24 pioneer hospitals had developed specialized centralized early rehabilitation units, on a small scale. But how can they be helped to thrive? According to experience in a very similar field from the developed world, well-functioning trauma systems are formed by major trauma centers and surrounding trauma networks. The concentration of major trauma in high-volume centers is key, but these centers must be adequately resourced to deliver a high-quality service (28). One interesting finding in our survey also indirectly suggests the importance of concentration of patients and resources: early rehabilitation of critically ill children is better developed in children's hospitals than in general hospitals or maternal and child health hospitals. We suspect two major reasons for this: firstly and most importantly, critically ill children are mostly admitted and treated in PICUs in children's hospitals, and the clinical need for early rehabilitation drives its development. Secondly, pediatric subspecialty development receives more attention and resources in children's hospitals. This suggests that the limited resources available should be concentrated to create regional specialized centralized early rehabilitation units and establish referral protocols for the transfer of patients from surrounding networks to these units.

Given the above, we suggest creating two-level integrated early rehabilitation systems that include specialized centralized early rehabilitation units and surrounding early rehabilitation networks within a region. Specialized centralized early rehabilitation units with professional multidisciplinary team could be built in the regional medical centers. When critically ill children having achieved medical stability in the ICU/PICU and with serious dysfunctions and comorbidities in need of a continuous chain of early rehabilitation, they can be transferred to the units to get not only the rehabilitation therapy to facilitate further functional recovery, but also the identification and management of the comorbidities to achieve a stable physical state for rehabilitation therapy. The surrounding early rehabilitation networks are ICU/PICUs at all types and levels of hospitals within the radiation range of specialized centralized early rehabilitation unit in the regional medical center. They should implement early mobilization and rehabilitation into everyday practice and transfer patients in need of further comprehensive early rehabilitation to the centralized early rehabilitation units. These units should be standardized and accept referral patients from surrounding networks. They should provide training opportunities to staff within their systems. From our practical experience, we have observed that there are critically ill children whose acute hospital needs have been met but who still have physical and cognitive disabilities. If the continuous chain of early rehabilitation is not available to them, the observation period and waiting time to transfer them out of the PICU is longer, which aggravates the patients' financial burden and delays the reunion of these children with their families. It also wastes resources and strains the PICU capacity for new patients.

## Limitations

Our findings should be interpreted within the context of several limitations. Firstly, as mentioned previously in Section Discussion, the survey was carried out within the framework of the Rehabilitation Group of the Pediatrics Branch of the Chinese Medical Association and only tertiary care centers were surveyed. We should not ignore that this has caused a concentration effect and we need to acknowledge that the reality is worse than the survey suggests, reinforcing the importance of developing early rehabilitation for critically ill children in China. Secondly, there may be respondent reporting bias because we adopted a self-report method. However, we tried to minimize this bias by limiting the questionnaire respondents to department directors, who should know their departments best. Also, the questionnaire was carefully designed, evaluated and revised before formal use.

## Conclusion

In conclusion, this survey revealed two notable facts: (1) early rehabilitation for critically ill children is not widely available in China; (2) only a minority of hospitals implement a continuous chain of early rehabilitation. These may result in many critically ill children not receiving the necessary standard early rehabilitation therapy in time, and the discontinuation of early rehabilitation is often inevitable for most patients during the referral period.

Given the urgency in China of developing a continuous chain of early rehabilitation for critically ill children, with limited resources, we suggest that a two-level integrated system is created which includes centralized early rehabilitation units and surrounding early rehabilitation networks within a region. ICU/PICUs at all types and levels of hospitals should be encouraged to implement early mobilization and rehabilitation into everyday practice and transfer patients in need of further comprehensive early rehabilitation to centralized early rehabilitation units.

Further research on the safety, efficacy and cost-effectiveness of centralized early rehabilitation units for critically ill children is needed.

## Data availability statement

The original contributions presented in the study are included in the article/Supplementary material, further inquiries can be directed to the corresponding authors.

## Author contributions

TZ designed the study, designed the questionnaire, analyzed the data, reviewed the literature, and wrote the manuscript. XD helped to design the questionnaire and draft the manuscript. YF, WJ, and XH helped to design the questionnaire. YC participated in the design of the study and the questionnaire. NX participated in the design of the study and the questionnaire and coordinate the study. All authors have made substantial contributions to this study and manuscript and participated in the data collection, manuscript revision, and approved the final manuscript.

## Conflict of interest

The authors declare that the research was conducted in the absence of any commercial or financial relationships that could be construed as a potential conflict of interest.

## Publisher's note

All claims expressed in this article are solely those of the authors and do not necessarily represent those of their affiliated organizations, or those of the publisher, the editors and the reviewers. Any product that may be evaluated in this article, or claim that may be made by its manufacturer, is not guaranteed or endorsed by the publisher.
