# Supplementary material for: Implementation of early rehabilitation for critically ill children in China: A survey and narrative review of the literature
Source: Front Pediatr. 2022 Aug 12;10:941669. doi: 10.3389/fped.2022.941669 (PMC9411939; doi:10.3389/fped.2022.941669)
Supplement: Supplementary file 1 [file Data_Sheet_1.docx]

**Appendix 1: English translation version of our questionnaire.**

**Survey on the implementation of early rehabilitation for**

**critically ill children in tertiary hospitals in China**

The investigation was conducted by the Rehabilitation Group of Pediatrics Branch of the Chinese Medical Association, aiming to understand the current status of implementation of early rehabilitation for critically ill children in tertiary hospitals in China. *Early rehabilitation* means “early initiation of rehabilitation interventions in the acute hospital, including in the ICU and/or in the acute care ward”. This survey is only for academic research, hoping to provide information for our academic group to make a work plan and to provide suggestions to sanitary administrative organs. The information obtained from the survey is completely confidential, with no specific unit data released, only the overall research results would be published.

If you agree to participate in the survey and answer the questions truthfully, it will be a great contribution to the development of rehabilitation medicine services in China. Thank you for your cooperation!

The person filling this questionnaire should be the director of departments in tertiary hospitals. All the questions in this questionnaire have multiple answers, please choose the most suitable answer/answers according to the current status of your department. Thank you.

| **General information** |
| --- |
| **Your name：**  **The name of your hospital：**  **Your department：**  **Your position：** |
| **The status of implementation and planning** |
| 1. Has your department implemented early rehabilitation for critically ill children?   A. Yes  B. No   1. If the answer to question 1 is no, is there any plan to implement early rehabilitation for critically ill children in the next 3 years?   A. Yes  B. No |
| **The form and scale of implementation** |
| 1. Has your department implemented early rehabilitation for critically ill children in ICU/PICU？ 2. Yes 3. No 4. Has your department built permanent specialized centralized early rehabilitation units for critically ill children？ (If the answer is no, the survey is over; If the answer is yes, please continue.) 5. Yes 6. No 7. The number of beds in the specialized centralized early rehabilitation units in your department：   (Fill in the blanks please)   1. The number of patients accepted by the specialized centralized early rehabilitation units per year.   A．less than 10  B．10-50  C．50-100  D．100-200  E．200-300  F．over 300 |
| **Treatment capacity** |
| 1. Types of diseases/injuries/conditions can be treated in your specialized centralized early rehabilitation units: (You may select one or more choices. ) 2. acquired neurological injury caused by infections, trauma, and other pathogenies 3. post nervous system tumor surgery 4. post epilepsy surgery 5. post orthopaedic surgery 6. post cardiac surgery 7. other diseases (Fill in the blanks please) 8. Type of dysfunction can be treated in your specialized centralized early rehabilitation units: (You may select one or more choices. ) 9. disturbance of consciousness 10. motor dysfunction 11. respiratory dysfunction 12. swallowing dysfunction 13. bladder/urethral dysfunction 14. other dysfunctions (Fill in the blanks please) 15. Supportive equipment in situ when patients are transferred to your specialized and centralized early rehabilitation units: (You may select one or more choices. )   A. Tracheal tube  B. Nasogastric tube  C. Jejunum tube  D. Indwelling catheter  E. Peripherally inserted central catheter  F. Central venous catheter  G. Ventilator  H. other (fill in the blanks please)   1. Indications for transfer out of rehabilitation unit: (You may select one or more choices. )   A. seizures occurring several times a day  B. seizures occurring >10 times a day  C. status epilepticus  D. pneumonia  E. severe pneumonia  F. respiratory failure  G. cardiac failure  H. intracranial hypertension  I. other (fill in the blanks please) |
| **Medical staff** |
| 1. Is there independent workgroup in your specialized centralized early rehabilitation units？(If the answer is D, the survey is over here; If the answer is A or B or C, please continue.)   A. we have independent workgroups comprising doctors, nurses and therapists  B. we have independent workgroup comprising doctors and nurses  C. we have independent workgroup of doctors  D. we have no independent workgroup   1. Doctor specialist of the highest proportion:   A. rehabilitation medicine  B. pediatric neurology  C. pediatric osteology  D. pediatrics/pediatric surgery  E. traditional Chinese medicine  F. general medicine  G. other majors (fill in the blanks please)   1. Doctor specialist of the second highest proportion:   A. rehabilitation medicine  B. pediatric neurology  C. pediatric osteology  D. pediatrics/pediatric surgery  E. traditional Chinese medicine  F. general medicine  G. other majors (fill in the blanks please)   1. Doctor specialist of the third highest proportion:   A. rehabilitation medicine  B. pediatric neurology  C. pediatric osteology  D. pediatrics/pediatric surgery  E. traditional Chinese medicine  F. general medicine  G. other majors (fill in the blanks please)   1. Therapist specialist of the highest proportion:   A. rehabilitation therapeutics  B. sports medicine  C. traditional Chinese medicine.  D. nursing  E. other majors (fill in the blanks please)   1. Therapist specialist of the second highest proportion:   A. rehabilitation therapeutics  B. sports medicine  C. traditional Chinese medicine.  D. nursing  E. other majors (fill in the blanks please)   1. Therapist specialist of the third highest proportion:   A. rehabilitation therapeutics  B. sports medicine  C. traditional Chinese medicine.  D. nursing  E. other majors (fill in the blanks please)   1. Departments in which doctors are available for consultation in your hospital: (You may select one or more choices. ) 2. ICU 3. Neurology 4. Neurosurgery 5. Respiratory 6. Orthopedics 7. Otolaryngology 8. Nutrition 9. Imaging department   I． other (fill in the blanks please) |
